# Swine clones: potential application for animal production and animal models

**DOI:** 10.1590/1984-3143-AR2024-0037

**Published:** 2025-01-20

**Authors:** Thaís Naomi Gonçalves Nesiyama, Juliano Rodrigues Sangalli, Tiago Henrique Camara De Bem, Kaiana Recchia, Simone Maria Massami Kitamura Martins, André Furugen Cesar de Andrade, Juliana Germano Ferst, Gustavo Henrique Doná Rodrigues Almeida, Mariana Groke Marques, Renata Gebara Sampaio Dória, Adriano Bonfim Carregaro, Marcus Antônio Rossi Feliciano, Maria Angélica Miglino, Fabiana Fernandes Bressan, Felipe Perecin, Juliano Coelho da Silveira, Lawrence Charles Smith, Vilceu Bordignon, Flávio Vieira Meirelles

**Affiliations:** 1 Faculdade de Zootecnia e Engenharia de Alimentos – FZEA, Universidade de São Paulo – USP, Pirassununga, SP, Brasil; 2 Faculdade de Medicina Veterinária e Zootecnia – FMVZ, Universidade de São Paulo – USP, Pirassununga, SP, Brasil; 3 Embrapa Suínos e Aves, Concórdia, SC, Brasil; 4 Faculté de Médecine Vétérinaire – FMV, Université de Montréal – UdeM, Montréal, Quebec, Canada; 5 McGill Faculty of Agricultural and Environmental Science – FAES, McGill University, Montréal, Quebec, Canada

**Keywords:** cloning, genetically modified swine, SCNT, oocytes, embryos

## Abstract

Somatic cell nuclear transfer (SCNT), or cloning, is used to reprogram cells and generate genetically identical embryos and animals. However, the cloning process is inefficient, limiting its application to producing valuable animals. In swine, cloning is mainly utilized to produce genetically modified animals. Indeed, recombinant DNA technologies have evolved considerably in recent years, with homologous recombination and gene editing technologies becoming more efficient and capable of recombining both alleles in a single cell. The selection of appropriate cells and their use as nuclear donors for SCNT is the most common method for generating edited and genetically modified animals for commercial and research purposes. This article reviews current applications of swine cloning and shares our personal experiences with the procedure in this species.

## Introduction

Even though animal cloning by somatic cell nuclear transfer (SCNT) remains an inefficient technique, it stands as an important reproductive biotechnology utilized by many laboratories worldwide. According to Campbell et al. (1996), animal cloning aims to intentionally produce a new animal through the nuclear transfer technique, using the nucleus of an animal tissue cell and an enucleated oocyte, enabling the replication of individuals with genetic material identical to the original.

Cloning studies have been carried out since the end of the 19th century. The word cloning comes from “Clons,” a specific group of plants capable of propagating asexually through different parts such as the tuber, stalk, bulb, shoots, branches, and grafts, transmitting identical characteristics to their descendants (Webber, 1903). In 1891, Hans Driesch produced two animals by separating the cells of a single sea urchin embryo by shaking them in a beaker of seawater (Sander, 1997). Conducting experiments with amphibians, Hans Spemann was able to replicate salamanders by embryonic bipartition using a strand of baby hair, being the first to propose the possibility of cloning through nuclear transfer (Spemann, 1938). The nuclear transfer (NT) technique was successfully carried out a few years later by German scientists who transferred the nucleus of a tadpole cell into a previously enucleated frog egg, albeit without producing offspring (Briggs and King, 1952). The first lived clone of differentiated cells was obtained by Dr. John Gurdon in Xenopus using a tadpole intestinal cell nucleus (Gurdon and Uehlinger, 1966).

Mammalian cloning was first reported in mice using embryonic cells (Illmensee and Hoppe, 1981) and subsequently in sheep clones (Willadsen, 1986). However, the breakthrough was obtained when Dolly, the first clone born from somatic cells, was produced from the nucleus of a mammary gland cell of an ewe, marking a milestone for biotechnology by demonstrating the reprogramming of differentiated cells from an adult animal into a totipotent zygote (Wilmut et al., 1997). Since then, clones from various animal species have been reported, such as mice (Wakayama and Yanagimachi, 1998), cattle (Kato et al., 1998), goats (Baguisi et al., 1999), pigs (Grisham, 2000), cats (Shin et al., 2002), rabbits (Chesné et al., 2002), mules (Woods et al., 2003), and horses (Galli et al., 2003), among others.

The integration of genetic manipulation with the SCNT approach used to produce Dolly has contributed to other biomedical research models, such as transgenic rabbits, sheep, mice, pigs, and other animals (Hammer et al., 1985; Colman, 1999). For instance, the transgenic sheep Tracy, produced by PPL Inc. at the Roslin Institute in Scotland, was the first to produce milk containing human alpha1 anti-trypsin, used in trials to treat cystic fibrosis (Wright et al., 1991). Additionally, Polly and Molly, other transgenic sheep produced by PPL, were genetically modified to produce human coagulation factor IX in milk (Schníeke et al., 1997). Nevertheless, even decades after the first report of cloned animals and its worldwide dissemination, SCNT remains an inefficient technique carried out in an artisanal manner by a well-trained embryologist, yielding only a small number of reconstructed zygotes (Alberio and Wolf, 2021). This manuscript intends to review the SCNT procedures, focusing on recent advances and applications in swine.

## The case for using the swine model

Pigs possess many advantages over other domestic mammals (Gutierrez et al., 2015). First, they are widely used for meat production and are prevalent worldwide. Their reproductive efficiency is particularly notable, with sows reaching sexual maturity in approximately five months. With a short gestation period of 114 days and an estrous cycle of 21 days year-round, these prolific animals typically produce between 12 and 16 piglets per parturition (Moya and Secco, 2021). Moreover, due to their polyovulatory characteristic, sow ovaries produce multiple antral follicles and a high number of oocytes can be obtained per aspiration for use in *in vitro* maturation (IVM), and *in vitro* embryo production (IVP) (Marques and Zanella, 2021). Currently, commercial pig reproduction primarily relies on artificial insemination (AI) using fresh semen, as freezing semen poses challenges in this species (Spate et al., 2013). For in vitro embryo production, a higher incidence of polyspermy following *in vitro* fertilization (IVF) represents a major challenge, with harmful consequences for embryonic development (Chen et al., 2021).

The production of genetically modified (GM) animals via SCNT requires cell and embryo biology expertise, including IVM of oocytes, culture of nuclear donor cells, nuclear transfer, parthenogenetic activation, and *in vitro* embryo culture (IVC). Synchronization and selection of recipients for reconstructed embryos are additional crucial steps for success in producing clones, genetically edited and transgenic animals. Despite the challenges involved, SCNT's production of pig embryos carries many positive implications. For example, a large number of swine slaughterhouses across the globe can provide substantial numbers of ovaries for oocyte collection. Moreover, the pig model for cloning allows large-scale production and transfer of embryos compared to other species. Due to the unique aspects of swine reproductive physiology, a single sow can serve as a recipient for 2-3 hundred reconstructed zygotes, thereby elevating the potential for clone production. Herein, we describe the swine SCNT technique and recent advances in various applications of genetically modified and cloned animals (Glanzner et al., 2023).

## The oocyte collection and *in vitro* maturation

The production of viable mature secondary oocytes from immature oocytes aspirated from antral follicles poses a significant challenge in swine IVP. Problems during this phase are usually related to acquiring oocyte competence for development. Although porcine oocytes reach blastocyst stages *in vitro*, success rates are still below those observed in cattle (Chen et al., 2021). However, studies in pig IVP, IVF, parthenogenesis, cloning, and embryo culture have progressed despite species-specific complexities. Oocyte morphology and physiology vary between species, impacting quality assessment. Maturation lengths also differ substantially among species, with approximately 22 hours for cattle, 30 hours for goats, 36 hours for horses, and 44 hours for pigs, requiring a split of the maturation protocol in IVM I and IVM II (Chaves and Figueiredo, 2010; Bordignon et al., 2013). Nuclear and cytoplasmic changes are needed during IVM to achieve oocyte developmental competence. Unfortunately, current IVP methods do not yield embryos of the same quality as *in vivo-*derived embryos, indicating the need for further protocol improvements. Since cumulus cells play a crucial role in completing meiotic maturation and fertilization, the co-culture of oocytes with cumulus cells and the addition of porcine follicular fluid (PFF) during IVP are frequently used in standard protocols attempting to mimic in vitro the physiological pathways of oocyte maturation occurring in vivo (Spate et al., 2014).

Typically, ovaries are collected from gilts (>150 days old) post-mortem at slaughterhouses, transported to the laboratory for immediate follicular aspiration at 34-36 °C in thermos bottles containing 0.9% saline solution, 75 mg/mL penicillin and 50 mg/mL streptomycin. Cumulus-oocyte complexes (COCs) are collected from antral follicles (3-6 mm in diameter) using 10 mL disposable syringes attached to 18-gauge needles. Follicular fluid aspirated from the ovaries is placed in 15 mL conical tubes and kept for 5 minutes for sedimentation. The recovered COCs, which present homogeneous cytoplasm and compact cumulus cells, are selected in Petri dishes, washed three times in TCM medium with HEPES, containing 20% of porcine follicular fluid (PFF), with 20µg/mL gentamicin, and transferred to IVM medium (Bordignon, 2019; Macedo et al., 2019). Selected COCs are *in vitro* matured in two steps. The first step, IVM I (0-22h), consists of TCM-199 medium (Gibco, BRL) supplemented with 20% (v/v) PFF from a single batch of 50 mL previously collected (stored in 1 mL aliquots), 0.91mM sodium pyruvate, 3.05mM D-glucose, 0.57mM L-cysteine, 10ng/mL EGF, 5 IU/ mL of human chorionic gonadotropin (hCG), 10µg/mL of equine chorionic gonadotropin (eCG), 1mM of cyclic AMP (cAMP), and 20µg/mL of gentamicin. The second step, IVM II (22-44h), uses the same medium as IVM I but lacks hormones (hCG, eCG) and cAMP. The oocytes were kept in four-well plates, groups of 50 COCs/per well, containing 500μl of IVM II medium. The COCs are maintained at 38.5 °C, in 5% CO_2_, and humidified air for 44 hours (Glanzner et al., 2023).

## The enucleation process and nuclear transfer

Cloning by SCNT is mainly used for commercial purposes to produce cattle and horses in countries such as the United States, Argentina, Brazil, China, and Italy, among others (Gambini et al., 2014; Maserati and Mutto, 2016). It is mainly employed to generate commercial or genetically valuable individuals, athletes, pet animals, and as a method for preserving endangered species (Hinrichs, 2006; Moro et al., 2015; Bordignon, 2019). It is also used to create genetically modified animals, which serve various purposes, including biomedical models for diseases, such as swine for xenotransplantation research, production of pharmacological proteins, improving genetic selection, and introducing new productive traits such as disease resistance in livestock (Wilmut, 1998; Alberio and Wolf, 2021).

A quarter of a century after the birth of Dolly the sheep (Wilmut et al., 1997), cloning technology still needs improvement, primarily due to low-efficiency rates (ranging from 0.1% to 15%). However, GM animals mostly rely on the SCNT technique to obtain the best results and keep the lineages alive (Alberio and Wolf, 2021). To produce GM animals, it is essential to apply cellular genetic editing tools, such as meganucleases, zinc-finger nucleases (ZNFs), and Tal-effector nucleases (TALENs). However, they are less efficient and more challenging to execute with precision when compared with the most recent and innovative technique, the Cluster Regularly Interspaced Short Palindromic Repeat (CRISPR)/cas9. The CRISPR-cas9 technique applies a gRNA oligonucleotide as well as an RNA-guided cas9 nuclease. Once the target site is recognized, the nuclease induces a double-strand DNA break with high efficiency (Oliveira et al., 2019).

Although, in theory, gene editing may be applied to IVF zygotes without creating mosaicism, few studies describe very efficient biallelic modifications without mosaicism (Mehravar et al., 2019; Yamashita et al., 2020). Therefore, most of the edited animals are generated by the association of the genetic editing tool with the SCNT method for cloning embryos (Jinek et al., 2012; Hryhorowicz et al., 2020; Tanihara et al., 2021; Whitworth et al., 2022).

**Figure 1:**
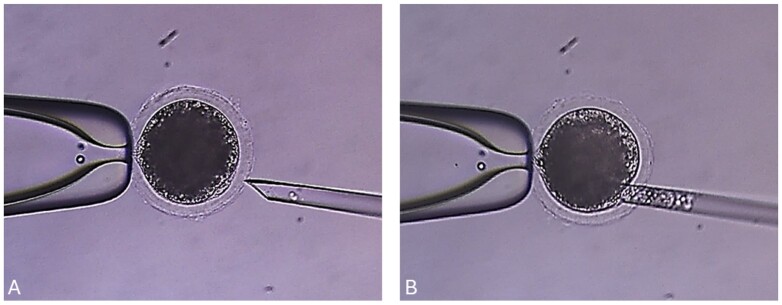
Illustration of the enucleation process for swine cloning using an inverted microscope equipped with a micromanipulation apparatus. (A) The matured pig oocyte is held in place by suction with the holding pipet (left side), while the injection pipet (right side) is positioned close to the first polar body; (B) Aspiration of the first polar body and adjacent ooplasm containing the metaphase plate without fluorescent DNA stain (Unpublished data).

Once genetically modified swine donor cells are selected, based on molecular confirmation of the GM and phenotype, the next step is deriving new cell cultures to continually clone this animal as a GM founder to ensure the birth of replicas (Alberio and Wolf, 2021). Therefore, the biotechnology to produce GM swine requires many *in vitro* matured oocytes and viable nuclear donor cells for SCNT (Lee and Prather, 2013; Whitworth et al., 2022). To produce embryos by SCNT, matured oocytes are denuded from the COCs and then enucleated in modified PBS containing 4 mg/mL BSA and 7.5 μg/mL cytochalasin B without calcium ions. The first polar body and the metaphase II (MII) spindle of the oocytes, which can be visualized following exposure to 0.4 μg/mL demecolcine and 0.05 M sucrose for 1 h, are removed with an enucleation needle (Figure 1). In swine, this enucleation process proved more efficient than removing the cytoplast close to the first polar body, and in confirmation of the enucleation process by epifluorescence after staining with Hoechst 33342 (Bordignon, 2019).

Swine somatic cells are individually isolated by trypsinization and used as nuclear donor cells by transferring them into the perivitelline space (Bordignon, 2019; Macedo et al., 2019). Donor cells are typically obtained by *in vitro* culture, usually from primary fetal fibroblast cultures grown to 90% confluence, although many different cell sources may be applied, for example, spermatogonial stem cells (Lee, et al 2019). Isolated donor cells are obtained by trypsinization at 0.25% for 5 minutes at 37ºC, followed by washing in cell culture medium, and centrifugation at 500 G for 5 minutes. The cell pellet is then resuspended in 1-2 mL of culture medium and added to the micromanipulation plate for SCNT use. In this protocol, cells must be at the G0-G1 cycle stage. However, other strategies may be applied, like using cells during the programmed cell death pathways (Miranda et al., 2012) or at any cell cycle phase if the recipient oocyte cytoplasm is at telophase II at enucleation (Bordignon and Smith, 1998). After the SCNT step, the oocytes and somatic cells are fused with a single DC pulse of 1.6KV/cm with 70μs in 0.28M mannitol medium containing 1μM CaCl2·2H2O, 0.05mM MgSO4. Reconstituted oocytes are then parthenogenetically activated, using 15Mm Ionomycin for 5 min, followed by exposure in 10μg/mL Cycloheximide, 7.5μg/mL Cytochalasin B, and 10mM Strontium for 4 h in porcine zygote medium 3 (PZM-3). After the activation, the embryos are washed and stay in *in vitro* culture (IVC) in PZM-3, in a humidified atmosphere of 5% CO_2_, 5%O_2_, and 90% N_2_, at 38.5 °C for 2 days, when cleavage rates are evaluated (Bordignon and Smith, 2008; Bordignon et al., 2013).

## The embryo transfer (ET) and pregnancy detection

Cloned embryos that cleave and have 2-4 cells at 24 to 48 hours after activation are transferred to recipient gilts (>180 days old). Co-transfer of parthenogenetic embryos can be performed to increase maternal-fetal recognition. Recipients chosen to receive the cloned embryos must be synchronized before ET using synthetic progesterone supplementation (32mg of Altrenogest® daily for 14 days). The selected recipient must show clear signs of estrus, e.g., swollen vulva and response to dorsal pressure. Furthermore, at the time of surgery, the animal must present the ovaries with early-stage *corpus luteum*, indicating that the female is at the proper cycle stage to receive 2-day-old cloned embryos at the 2 to 4-cell stage (Macedo et al., 2019; Glanzner et al., 2023).

**Figure 2:**
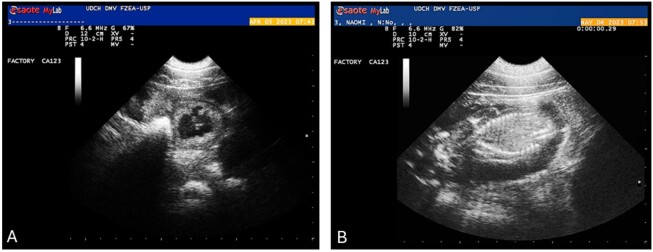
Echography images of a SCNT pregnancy. (A) Image of a transabdominal echography showing a gestational vesicle of cloned minipig at 33 days after ET (April 05, 2023), and (B) a formed cloned fetus at 62 days after ET (May 04, 2023) (Unpublished data).

The ET is performed into both uterine horns through laparotomy under general anesthesia using the ampoule opening approach. Post-surgical care is necessary for the following 72 hours. Pregnancy is confirmed by ultrasound on the 25^th^ day post-ET, and the female is monitored daily during the pregnancy, with a set of digital cameras being a recommended option, until the expected delivery date, while receiving uninterrupted supplementation of synthetic progesterone (24 mg of Altrenogest® daily) until parturition. Regular ultrasound monitoring of pregnancy progress can be conducted, for example, on days 35, 45, 55, 65, 75, 90, 100, and 110 (Figure 2). The chances of conceptuses survival increase after 60 days, after which they surpass the most critical phase of fetal loss (Lee and Prather, 2013).

**Figure 3:**
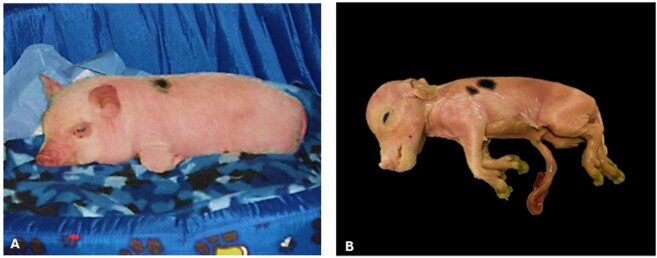
Somatic cell donor animal and cloned fetus. (A) Image of the original minipig from which donor cells were obtained (~1 month old); (B) Image of the stillborn cloned minipig conceptus on the day of cesarian section (at 114 days of gestation) obtained after 140 SCNT embryos and 22 parthenotes ET, distributed in both oviducts. The stillborn clone measured 196.84 mm crown-rump and weighed 320g (Unpublished data).

**Figure 4:**
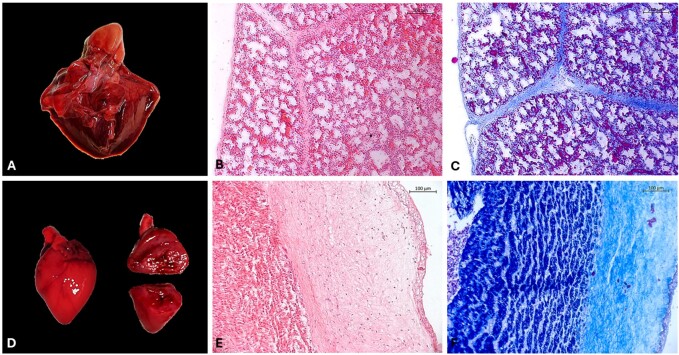
Macroscopic and histologic images of the cloned minipig lungs and heart showing apparently normal morphology. Macroscopic image of the lungs (A) and heart (D). Histologic images of the lungs stained with Hematoxylin and Eosin (B) and Masson stain (C). Histologic images of the heart stained with Hematoxylin and Eosin (E) and Masson stain (F). Images were acquired at 10x magnification (Unpublished data).

Progesterone supplementation is suspended 2-3 days before the expected delivery date to align with normal physiology, as progesterone must fall to initiate labor. Furthermore, to closely monitor signals at parturition around 114 days, a final ultrasound examination may be performed to evaluate fetal heartbeat before the cesarean section. In the event of the death of one or more fetuses, images for morphometric data, weight, size (crown-rump) (Figure 3), and biological materials, such as organs (Figure 4), blood, and placental tissues, can be collected for further investigation. Photos of the fetuses are also valuable for comparison with the phenotypic characteristics of the original nuclear donor animal (Glanzner et al., 2023; Siriboon et al., 2014).

## The peri-parturition and neonatology demand

During pregnancy, the fetus and all placental tissues should develop normally, resulting in adequate nutrition and the birth of a healthy conceptus (Wilsher and Allen, 2011). SCNT was used to clone several species over the years, and many live animals have been obtained. However, cloning presents reprogramming errors and failures, leading to problems in implantation and fetal development (Cho et al., 2007; Arnold et al., 2008). Unfortunately, little is known about the origin of these embryonic problems. Yet, they are also associated with neonatal and placental problems, which have been observed in several animal species, such as cattle, horses, and pigs, among others. Placental abnormalities can also impact birth rates and offspring viability (Bordignon and Smith, 2008).

Problems such as dystocia, retained placenta, and hydroallantois are frequently observed in clone pregnancies in other farm species (Palmieri et al., 2008; Chavatte-Palmer and Tarrade, 2016). The amniotic and chorioallantoic membranes, with a high incidence, are abnormally enlarged with gross edema. Additionally, the umbilical cords may be abnormally edematous and engorged, failing to rupture naturally and sometimes requiring the use of hemostatic clamps or even surgical intervention to prevent bleeding (Chavatte-Palmer et al., 2002; Johnson and Hinrichs, 2015). Newborn swine clones are more vulnerable than counterpart newborn pigs conceived naturally and frequently experience cardiopulmonary problems (Siriboon et al., 2014). They often require intensive postpartum support and care, such as oxygen therapy, fluid therapy, and medication. Immediate assistance to newborns is vital for the survival of cloned piglets. Whether the clones are genetically edited or not, they represent significant investments, making it essential to understand the causes of these complications and pregnancy losses (Siriboon et al., 2014; Pozor et al., 2016).

## Are genetically modified swines available and approved for commercial animal use?

Breeding and animal selection are based on finding genes or alleles with favorable characteristics for selective reproduction. However, the transmission of the desired trait depends on the heritability of these characteristics, often involving complex polygenic interactions. With the global population demand rising and environmental concerns escalating, there is a pressing need to enhance animal production and agriculture while optimizing land resources (Whitworth et al., 2022). Genetic engineering technologies have emerged as a promising tool for genetic improvement offering unique opportunities for animal selection and introducing new traits when combined with AI and IVP (Tanihara et al., 2021). Notable examples are GM sows, which produce 70% more milk than control sows, resulting in heavier piglets (Noble et al., 2002). Also, the insertion of mouse genes into pigs has been utilized to regulate body temperature and fat metabolism, resulting in 24% less fat depositing than in normal animals. This could be a solution to produce more healthy pork meat in the future, reducing the facility's maintenance temperature costs and spending less energy (Zheng et al., 2017).

Additionally, gene editing enabled the production of commercial pigs resistant to diseases such as PRSSV (porcine reproductive and respiratory syndrome virus), CSFV (classical swine fever virus), and TGEV (transmissible gastroenteritis virus). Although challenges remain for diseases like influenza (Salvesen et al., 2022), efforts are ongoing to develop resistance against PEDV (porcine epidemic diarrhea virus), ASFV (Africa swine fever virus), and PRV (pseudo rabies virus) (Yuan et al., 2022). Disease-resistant animals are not restricted to viruses, as models related to other microorganisms are also included in the study (Singh and Ali, 2021). In late 2020, the United States Food and Drug Administration department approved the first gene-edited pig for commercial and biomedical use, the “Galsafe” pigs, which lack the alpha-gal sugar in their cell surfaces. This alteration was developed to avoid hyperacute rejection in xenotransplantation but also reduces immunological response (allergies) to pork meat consumption (Dolgin, 2021). All these examples of gene-target animals were made by modifying the genome using different molecular tools and SCNT (Prather et al., 2013; Chavez et al., 2023).

## Examples of swine models used for biomedical research

Since ancient Greece, pigs have been used as an experimental model in biomedical research due to their physiological, anatomical, and genetic similarity with humans (Gutierrez et al., 2015; Hryhorowicz et al., 2020; Tanihara et al., 2021). Also, because pigs have a metabolism, size, and longevity similar to humans, they are better models for human research diseases than other species, such as small primates and mice (Alberio and Wolf, 2021). Genetically engineered (GE) pigs have been produced for many studies (Carrier et al., 2022), such as hormonal diseases like diabetes (Zettler et al., 2020), neurodegenerative disorders (Yang et al., 2021), cystic fibrosis (Caballero et al., 2021), dystrophies (Moretti et al., 2020), cardiovascular diseases (Schneider et al., 2020), and human cancer studies and treatments, such as the Onco-pig (Schook et al., 2015). These models closely mimic the mechanism of human diseases.

## Applications towards the xenotransplantation

Demands for organ transplantation, such as kidneys, lungs, heart, liver, pancreas, or other, due to disease or accident entail enduring lengthy waiting lists. Hence, the evaluation of organ transplantation recipients and donors involves assessing age, health conditions, and other diseases, among other protocols and approved rules, determining their rank among other candidates or rejection. Data shows that the list of people in need of organ transplantation in the United States increases by 1% per year. In comparison, donors decrease by 1-4% annually, highlighting a genuine concern and a global organ shortage crisis (Alberio and Wolf, 2021). Besides extensive selection protocols, recipients for allotransplantation (from human to human, same species) must pass several tests and receive multiple immune suppressive drugs to decrease the immunological response. A more crucial immunological body response occurs to xenotransplantation (from animals to humans, different species) for various reasons, such as different types of cells, proteins, and carbohydrates. Furthermore, viruses in other organisms may also pose a risk. The shortage of human donors and ethical concerns have encouraged several institutes and their researchers to make considerable efforts to modify the pig’s genome. This effort resulted in pigs lacking molecules related to immediate immune response and, more recently, endogenous virus (Denner, 2022).

One prevailing example of xenotransplant in humans is the use of heart valves obtained from cows and pigs. These valves are processed by glutaraldehyde-fixed bioprosthetic heart valves (GBHVs), rendering them immunologically inert and improving outcomes (Manji et al., 2014). Furthermore, several trials have been performed in which gene-edited pig organs were transplanted to non-human primates. For example, pig heart transplantation to baboons allowed survival for up to 200-900 days (Mohiuddin et al., 2014). Other pig organs like kidneys or skin were also xenotransplanted to baboons with a similar success rate (Tena et al., 2017; Iwase et al., 2017). Moreover, GM pig organs were xenotransplanted into humans in a few clinical trials. In 2021, a pig kidney was xenotransplantated to a brain-dead 50-year-old man, with the gene-edited pig having 10 genes modified (Cooper et al., 2021). The first gene-edited pig heart was xenotransplanted in 2022 to a 57-year-old man who was in a terminal stage of heart disease; this individual survived nearly two months after the transplant surgery (Wang et al., 2022).

**Figure 5:**
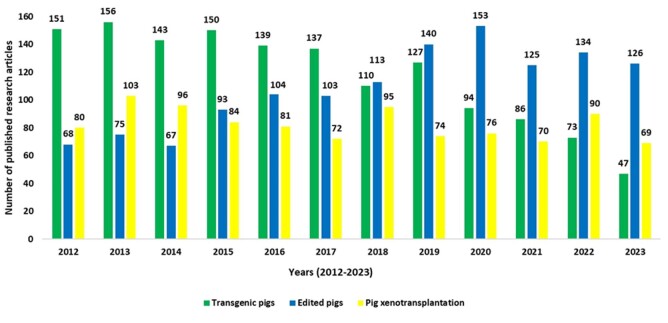
Number of research articles obtained in the Clarivate™ (Web of Science™) database in the last 11 years (2012-2023) related to genetic modifications in swine. The search was done with the keywords: “transgenic pigs,” “edited pigs,” and “pig xenotransplantation”. Overall, the number of research articles containing the keyword “transgenic pigs” decreased, while the number of publications mentioning “edited pigs” increased, possibly because of the CRISPR/cas9 technique efficiency. However, the keyword “pig xenotransplantation” did not change substantially, indicating a shift in the methodology for producing GM pigs for organ donation.

The new gene editing methodologies increased the potential of genome manipulation, resulting in the creation of many newly edited animals and advances, including reports of long-term survival in non-human primates and the first trials of xenotransplantation of pig organs to humans. A search on the Web of Science site in January of 2024 for the keywords “transgenic pigs”, “edited pigs”, and “pig xenotransplantation” indicated that since the discovery of the CRISPR cas9 technique in 2012, the number of published research articles about transgenic pigs has reduced to approximatively 1/3, from 151 in 2012 to 47 in 2023. On the other hand, the number of published research articles about edited pigs has almost doubled, from 68 in 2012 to 126 in 2023. Moreover, the number of published research articles mentioning pig xenotransplantation shows continuous interest in the last 12 years, ranging from 69 in 2023 to 103 in 2013 (Figure 5).

## Conclusion

This article describes and rationalizes the use of SCNT technology for generating genetically identical animals and outlines some of the reasons for cloning applications so far. Historically, most cloned animals born in the late 20^th^ century were related to producing high-value commercial animals. More recently, with the advent of new gene editing methodologies, there has been an increase in the efficiency and precision of genetic modification, consequently driving the demand for SCNT cloning. Due to their importance in animal production and as animal models for research, swine became very in demand. Pigs are preferred over primates and mice due to availability, ethical aspects, and physiological similarities to humans. Therefore, GM swine has emerged as promising candidates for biomedical research. As highlighted, genetic modifications in pigs, especially for traits related to healthier meat and disease resistance, are increasing, and receiving approval from regulatory agencies. In this scenario, SCNT, especially in swine, may have a longer list of contributions in the future. Due to these attributes, we predict that swine will soon become the most frequently cloned species.
